# Application of an Adapted Health Action Process Approach Model to Predict Engagement With a Digital Mental Health Website: Cross-Sectional Study

**DOI:** 10.2196/57082

**Published:** 2024-08-07

**Authors:** Julien Rouvere, Brittany E Blanchard, Morgan Johnson, Isabell Griffith Fillipo, Brittany Mosser, Meghan Romanelli, Theresa Nguyen, Kevin Rushton, John Marion, Tim Althoff, Patricia A Areán, Michael D Pullmann

**Affiliations:** 1 Department of Psychiatry and Behavioral Sciences University of Washington School of Medicine Seattle, WA United States; 2 Merck Upper Gwynedd, PA United States; 3 School of Social Work University of Washington Seattle, WA United States; 4 Mental Health America Alexandria, VA United States; 5 Allen School of Computer Science & Engineering University of Washington Seattle, WA United States; 6 NIMH Bethesda, MD United States

**Keywords:** Health Action Process Approach (HAPA), digital health, health behavior, Mental Health America (MHA), digital mental health engagement, mental health website

## Abstract

**Background:**

Digital Mental Health (DMH) tools are an effective, readily accessible, and affordable form of mental health support. However, sustained engagement with DMH is suboptimal, with limited research on DMH engagement. The Health Action Process Approach (HAPA) is an empirically supported theory of health behavior adoption and maintenance. Whether this model also explains DMH tool engagement remains unknown.

**Objective:**

This study examined whether an adapted HAPA model predicted engagement with DMH via a self-guided website.

**Methods:**

Visitors to the Mental Health America (MHA) website were invited to complete a brief survey measuring HAPA constructs. This cross-sectional study tested the adapted HAPA model with data collected using voluntary response sampling from 16,078 sessions (15,619 unique IP addresses from United States residents) on the MHA website from October 2021 through February 2022. Model fit was examined via structural equation modeling in predicting two engagement outcomes: (1) choice to engage with DMH (ie, spending 3 or more seconds on an MHA page, excluding screening pages) and (2) level of engagement (ie, time spent on MHA pages and number of pages visited, both excluding screening pages).

**Results:**

Participants chose to engage with the MHA website in 94.3% (15,161/16,078) of the sessions. Perceived need (β=.66; *P*<.001), outcome expectancies (β=.49; *P*<.001), self-efficacy (β=.44; *P*<.001), and perceived risk (β=.17-.18; *P*<.001) significantly predicted intention, and intention (β=.77; *P*<.001) significantly predicted planning. Planning was not significantly associated with choice to engage (β=.03; *P*=.18). Within participants who chose to engage, the association between planning with level of engagement was statistically significant (β=.12; *P*<.001). Model fit indices for both engagement outcomes were poor, with the adapted HAPA model accounting for only 0.1% and 1.4% of the variance in choice to engage and level of engagement, respectively.

**Conclusions:**

Our data suggest that the HAPA model did not predict engagement with DMH via a self-guided website. More research is needed to identify appropriate theoretical frameworks and practical strategies (eg, digital design) to optimize DMH tool engagement.

## Introduction

### Background

Access to mental health support is poor, with barriers including mental health stigma and limited time, availability, and accessibility [1]. According to the National Institute of Mental Health, it was estimated that 57.8 million adults in the United States lived with a mental illness in 2021 [2]. Digital Mental Health (DMH) is seen as the next generation of mental health care, in that it is readily accessible to most, affords anonymity when appropriate, and can be used when a consumer needs it, rather than when a provider is available. It serves a particularly valuable role where services are scarce, with studies finding disproportionate use of online mental health screening tools in rural areas [3].

In general, engagement with DMH is poor [4-7]. When using mobile apps, consumers in 1 study disengaged in as little as 2 weeks [8], and another study found a median 15-day retention rate of 3.9% [9]. However, there is limited research on engagement with mental health focused websites, which likely have different usage patterns than other approaches to DMH, such as mobile apps or digitally mediated therapy. Mental health websites are typically free, usually used for short-term information gathering, and may include a wide variety of self-guided resources such as psychoeducation articles, self-administered screening tools for common mental health problems, links to find therapists, downloadable self-monitoring tools (eg, medication management charts), ways to connect with peer support groups, and interactive self-help tools (eg, cognitive reframing activities using artificial intelligence). One website developed and maintained by Mental Health America (MHA) [10] features all of these DMH resources contained within approximately 500 pages, is free and openly accessible, and is visited by 9-10 million people a year, with 6.5 million using self-administered screening tools and 3.5 million accessing other resources. However, resources other than screening tools are underutilized. In particular, those who self-administer mental health screening tools are unlikely to engage with the other valuable resources on the website. For instance, in 2021, only 70% of those who used the website to self-administer a screening tool visited a nonscreening-related page on MHA. Only 15% subsequently read any articles despite the valuable psychoeducation and other materials available that could help them improve their emotions and behavior (Mental Health America, unpublished data, January 2024).

We partnered with MHA to conduct collaborative research with the goal of increasing optimal engagement with DMH resources on their website. We defined optimal engagement with DMH as engaging with resources that are aligned with users’ needs and interests and motivate users toward taking positive mental health actions. The Health Action Process Approach (HAPA) is a theoretical framework aligned with this goal. The HAPA model has been described in detail elsewhere [11-13], but here we describe it as applied to DMH tool engagement. The HAPA model is separated into 2 phases: *motivational* (when *intentions* are developed) and *volitional* (when *health actions* occur). During the motivational phase, preintention factors shape behavioral intention. Several mechanisms operate to support or hinder progression through the HAPA stages of health behavior change. Movement from preintention to intention is influenced by *perceived need* to engage in a health action [14], *task self-efficacy*, the degree to which consumers believe that they can engage in behavior change, *perceived risks* of not acting, and *outcome expectancies*, the positive and negative anticipated consequences of engaging in a behavior. If consumers have a high perceived need for change, high task self-efficacy, positive outcome expectancies of engaging with a DMH tool, and high perceived risks of not acting, they are more likely to move through the 2 stages of the motivational phase: *preintention* to *intention*. If task self-efficacy, perceived needs and risks, or outcome expectancies are low, then the consumer will be unlikely to set intentions. The volitional phase describes the process by which a consumer moves through the action stages: *behavioral intention* to *planning* to *acting* (ie, implementing a health behavior; in this case, engaging with DMH). Consumers with high intentions are more likely to implement a health behavior if they engage in planning. Other mechanisms in the traditional HAPA model include *maintenance* and *recovery self-efficacy* (the belief in the ability to continue a health behavior in the face of challenges, or restart if stopped) and *coping planning* (plans for how to continue an action in the face of challenges). These mechanisms are less relevant for the current research because they are focused on ongoing maintenance of behavioral actions. The HAPA model has robust empirical support in predicting health behavior change, such as dietary behaviors and medication adherence [15,16]. For example, 1 study found that medication adherence was significantly predicted by intention, task self-efficacy, coping self-efficacy, and coping planning in a sample of patients with type 2 diabetes [16].

We aimed to assess the applicability of the HAPA model to DMH engagement on a mental health website. We conducted the current research to assess whether the HAPA model fit our data and to examine the strength with which HAPA model constructs (eg, self-efficacy, perceived need, and perceived risks) were related to DMH engagement. This information would be used to develop and test tailored engagement strategies that match stage (eg, preintention, intention) and mechanism (eg, self-efficacy, perceived need) to the individual to improve DMH engagement. Tailored nudges (ie, strategies using a cue, such as a message, to influence user behavior) using this language could be developed and tested to help consumers move through the stages of behavior change (ie, from preintention or intention into the volitional stage of acting) to act on health-promoting behaviors (eg, through self-help tools, therapy, and building social connections). For instance, upon completing a screening tool, an MHA website visitor who endorses low self-efficacy could be shown language intended to increase self-efficacy to thereby increase their motivation to transition from self-administering screenings to initiating self-help or professional treatment.

There is precedent for applying the HAPA model to develop stage-matched interventions. Lippke et al [17] found that for intenders, but not preintenders, a physical activity planning intervention produced changes in intention and planning which were subsequently associated with increased physical activity. Self-efficacy, which was not targeted by the intervention, was not impacted by the intervention and not associated with physical activity. The authors concluded that interventions will be more successful when tailored to the participant’s stage of change, with intenders being more receptive to volition-based interventions as predicted by the HAPA model. Similar findings have been found when developing interventions tailored to stage of change and HAPA mechanisms. A study on hand hygiene behavior in hospital units found that interventions tailored to empirically assessed HAPA components (eg, providing information on the risks of not acting for units that scored low on risk perceptions) were associated with a decrease in infections, whereas units that received untailored interventions did not experience a decrease in infections [18].

Previous work has described how the HAPA model can be adapted for ongoing digitally delivered psychotherapy [19], but the use of DMH self-guided websites is likely to have different engagement patterns and associations. Website usage is likely to be more sporadic, short-term, and used by people in the preintention and intention stages, as they are determining what kinds of actions they want to take. In addition, the operationalization of a health action on a DMH website includes actions such as clicking on a navigation page, reading an article about depression, or using a web-based single-session intervention. Planning a health action in this context may be a seamless and instantaneous process that is difficult to separate from intention. Intention to act, planning, and action (clicking a link) may occur within a time span ranging from seconds to minutes, rather than days to weeks as in usual applications of the HAPA model [12,20-25]. Furthermore, it is unclear whether or how principles of marketing (strong call to actions) or user design (color and placement) determine user behavior as compared with the HAPA model.

A meta-analysis of 95 studies using the HAPA model [13] provides a summary of empirical literature applying the HAPA model to a wide variety of health behavior outcomes, such as dietary behavior and physical activity [26]. Self-efficacy and outcome expectancies were moderately correlated with intention, and risk perception effect sizes were small but statistically significant. In the meta-analytic path analyses, self-efficacy, intention, and action planning significantly predicted outcomes. Due to the small and nonsignificant role of risk perception, the authors suggested that this mechanism may play only a minor role in predicting health behaviors.

Only 3 (3.2%) of the studies included in the meta-analysis used an observed behavioral measure. The remaining studies used self-report measures of outcomes, and none of the studies examined DMH. Because using self-report measures for all HAPA constructs and outcomes is likely to inflate associations due to method bias, observable behavioral outcomes are necessary to reduce these biases [27]. In addition, most studies had a greater than 4-week time lag between the measurement of HAPA constructs and behavior. Whether the HAPA model is relevant for behaviors that occur with lags of seconds or minutes rather than weeks or months remains unknown.

An additional study not included in the meta-analysis examined the HAPA model in a DMH context and included both self-report and observed measures of engagement [14]. This study of engagement with a web-based intervention for trauma had mixed findings: self-reported engagement was associated with HAPA motivational constructs but observed engagement was not. These findings support the idea that self-reported engagement is more strongly associated with self-reported HAPA constructs than objective measures and that planning may be most effective for those with low self-efficacy.

In sum, several gaps exist in the HAPA model literature. Studies have relied primarily on self-report measures, with so few including observed or objective measures that a moderator comparison of self-report and objective measures could not be conducted in the meta-analysis. In addition, no studies applying the HAPA model to DMH were included in the meta-analysis because so few exist. We are aware of only 1 previous study that applied the HAPA model to DMH, which found attenuated effects when using an objective measure for engagement behavior [14]. Therefore, more research is needed to determine the strength of the HAPA model in a DMH context and which uses objective measures of behavioral outcomes.

### Aim of This Study

The purpose of this cross-sectional study was to test whether the HAPA model predicted DMH engagement and to determine which HAPA model constructs were most strongly associated with DMH engagement. In doing so, this manuscript contributes to the broader literature on the HAPA model by focusing on DMH engagement, employing an objective measure of health behavior, and measuring outcomes within seconds or minutes between the motivational and volitional phases of the HAPA model. In addition to contributing novel insight toward understanding engagement with DMH via the HAPA model, these findings could be applied to strategies to increase engagement by focusing on HAPA constructs in website design.

## Methods

### Participants

Data were collected via voluntary response sampling. Participants were a naturalistic sample of people who self-administered a mental health screener on the MHA website between October 2021 and February 2022*.* The inclusion criteria were clicking on the “Next Steps” survey and completing it for their own needs (as opposed to reporting that it was completed for others, such as their children). The exclusion criteria were completing less than 70% of the Next Steps survey or taking the “parent test” or “youth screener” (Pediatric Symptom Checklist [28,29]); there was no age restriction. The final analytic sample included data from 16,078 sessions (15,619 unique IP addresses from US residents) on MHA.

### Procedure

Users of the MHA website can self-administer any of several evidence-based mental health screening measures for depression, postpartum depression for new and expecting parents, anxiety, attention-deficit/hyperactivity disorder, psychosis or schizophrenia-spectrum disorders, posttraumatic stress disorder, eating disorders, substance or behavioral addictions, and youth mental health (separate versions for youth or caregivers to complete). Participants could click on any of these tests and take the online screener; for example, clicking “Depression Test” showed users the Patient Health Questionnaire-9 [30]. This study focuses on data collected from the Next Steps survey, which was an option participants could choose among these screeners and was also provided via a link on the results pages for screening measures. Participants could complete as many screeners as desired. After completion of each screener, including the Next Steps survey, the participants were shown an optional demographics survey. Data submissions were chunked into sessions, which were defined as all website interactions via a unique IP address bounded by a gap of at least 30 minutes.

### Ethical Considerations

All materials and procedures for this study were approved by the University of Washington Institutional Review Board (STUDY00010958). All data were protected under the University of Washington Institutional Review Board. Data sharing between MHA and the University of Washington followed a Data Security Protocol, which required data to be deidentified via random identifiers and stored securely on an encrypted institutional server accessible only by authorized research team members. The study’s external Data Safety and Monitoring Board oversaw study progress and participant safety. The Institutional Review Board and Data Safety and Monitoring Board determined that there were no anticipated risks for participants and no further application for ethical approval was required.

### Measures

#### Demographic Survey

The demographic survey included items about age range, race, ethnicity, and gender identity. A checkbox was provided for participants to indicate whether they identify as transgender. If participants completed more than 1 demographic measure during a session and these values differed, which was rare (<3%), we report “more than 1 answer given,” which should be distinguished from “more than 1 of the above.” For instance, an IP address that completed only 1 demographics measure and endorsed “Asian” and “Black” was categorized as “more than 1 of the above,” whereas an IP address that reported “Asian” on 1 demographics measure and “Black” on another screener was categorized as “more than 1 answer given.” It is possible that multiple people (eg, in the same household) may have completed the surveys from the same IP address.

#### Next Steps Survey

The Next Steps Survey was developed by the research team at the University of Washington collaboratively with MHA (Multimedia Appendix 1). Because this was a naturalistic study and participants were not incentivized to participate, the team aimed to use measures that were pragmatic, brief, tailored to the website activities, and consistent with MHA’s values and approach. No existing measures were suitable, as studies using the HAPA model often apply individualized items due to the study-specific nature of the constructs. Therefore, items were developed by the study team by adapting existing items developed and tested in other studies of the association between HAPA constructs and behavior [13,14,20,22,31-34]. The resulting 22-item survey designed to assess HAPA model constructs in the context of DMH consisted of four 5-item subscales (outcome expectancies, intention, self-efficacy, and planning) and 2 single items for perceived risk and perceived need. The 5-item subscales comprised questions that asked about the five types of resources available on the MHA website: (1) learning more about mental health (LMH), (2) connecting with others (CO) who have mental health conditions, (3) learning about treatment options (LTO), (4) receiving mental health treatment (RMH), and (5) using online self-help tools (ONL).

Outcome expectancies were measured by participant ratings on their belief that the 5 types of resources would improve their mental health on a 4-point scale ranging from 0 (*Definitely won’t help*) to 3 (*Definitely will help*). Intention items assessed whether participants intended to act on the 5 resources on a scale of 0 (*I definitely will not do this*) to 3 (*I definitely will do this*). Self-efficacy items asked participants to rate their confidence in their ability to act on the 5 items on a scale of 0 (*Not at all confident*) to 3 (*Very confident*). Planning items were yes/no questions that asked participants whether they had a specific plan to take any of those actions.

Perceived need was measured on a scale of 0 (*No*), 1 (*I don’t know*), and 2 (*Yes*). Perceived risk was measured on a 4-point scale from 0 (*No, it will get better on its own*) to 3 (*Yes, it will get much worse*). If participants completed more than 1 Next Steps survey during a single session, we report on data provided during the first completion within that session.

#### Engagement With Web-Based Content

Choice to engage was a dichotomous outcome indicating that participants visited any web pages, excluding screening pages. To correct for extremely brief web page visits that did not represent true engagement, based on literature that shows that users evaluate website design within seconds [35], we defined engagement as requiring at least 3 seconds on the MHA website excluding time spent on screening pages.

We operationalized level of engagement with 2 indicators: number of pages visited, or engaged pages (EP), and total engaged minutes (EM). EP was computed by summing the total number of web pages visited on the MHA website excluding screening pages. EM was computed by summing the total amount of time spent on MHA’s website excluding time spent on screening pages. Because time spent per page was computed from when a page on MHA was loaded to the time participants clicked on a link to another page on MHA, time spent on the final page viewed (eg, before closing a browser or visiting another website) could not be included in EM.

### Analyses

The goals of these analyses were to examine whether the HAPA model predicted engagement with web-based resources and which HAPA constructs were significant predictors of website engagement. We did this by testing the model fit of the adapted HAPA model to our data using structural equation modeling (SEM).

Data were collected from 21,329 sessions in which participants took the Next Steps survey. Of these, 75.9% (16,179/21,329) completed the Next Steps survey for their own needs and did not take the Pediatric Symptom Checklist. Analyses included data from only those participants who completed at least 70% of the 22 Next Steps survey items (N=16,078). Because participants completed the Next Steps survey during each session and may have exhibited different engagement patterns after ≥30 minutes of inactivity on the website, IP addresses with multiple sessions (n=605) were retained in the analysis, with the number of sessions for any 1 IP address ranging from 1 to 10. Response categories with less than 10% endorsement were combined with the next adjacent response category for accurate parameter estimation. Following collapsing of response categories, proportions of endorsement for each of the Next Steps survey items were no greater than 58.9% for any 1 response option.

**Figure 1:**
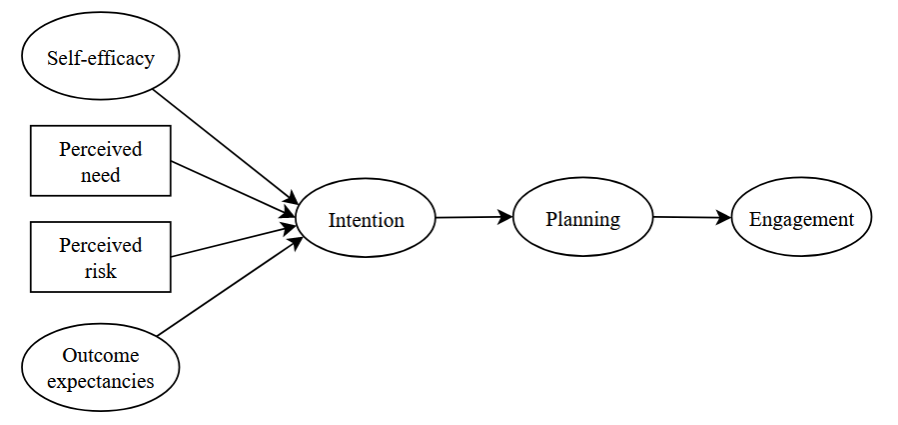
Hypothesized structural equation model based on the adapted Health Action Process Approach model.

Prior to SEM, we examined correlations between the Next Steps survey items, EM, and EP. We used the weighted least squares means and variance–adjusted (WLSMV) estimator, which computes a tetrachoric or polychoric correlation matrix using pairwise complete observations. Next, measurement models using unidimensional confirmatory factor analysis (CFA) were assessed for each reflective latent construct (self-efficacy, outcome expectancies, intention, and planning) using model fit statistics, with factor loadings as the parameters of interest. The measurement model for level of engagement was underidentified and model fit indices could not be estimated. The adapted HAPA model was tested by incorporating the HAPA constructs and their measured variables to form the theorized model (Figure 1). This model excludes maintenance self-efficacy, recovery self-efficacy, and coping planning, as these constructs are not relevant to individuals engaging with a website during a single session. Maintenance self-efficacy refers to one’s belief that they can overcome barriers to taking a health action, and coping planning refers to identifying methods to cope with those barriers. These constructs were excluded because there were no foreseeable barriers to the health action (ie, clicking a link) in the context of this study. Recovery self-efficacy refers to restarting a health behavior once it ends, which in our study would be returning to the website after usage. This is less relevant to our study as our research question is focused on an immediate behavioral outcome.

The first model predicted choice to engage, and the second analysis predicted level of engagement for those who did engage. All parameters were modeled and allowed to freely vary, and models were assessed by examining model fit indices and path coefficients. EM was modeled as a censored variable to account for the unrecorded time spent on the final page visited. Assumptions of multivariate normality and linearity were assessed in MPlus. While SEM is robust to nonnormality, recommended values of acceptable skewness range from –2 to +2, and acceptable values of kurtosis range from –7 to +7 [36,37]. Skewness and kurtosis for both EM and EP were well out of the acceptable range (EM skewness=5.19, kurtosis=40.58; EP skewness=5.74, kurtosis=92.56), indicating nonnormal distributions. The highest proportion of values was at 0 (5.6%) for EM and 2 for EP (13.6%; Table 1). The WLSMV estimator is used to analyze ordinal response data, does not require the observed variables to be normally distributed, and assumes that the underlying latent response variable is normally distributed [38-41].

**Table 1:** Ranges, means, variances, and percentage of zeros for engaged minutes and engaged pages.

			Range		Mean		Variance		Percentage of zeros
**Engaged minutes**									
	Full sample^a^	0-167.72		4.30		114.53		5.55^b^	
	≥3 seconds^c^	0.05-167.72		4.56		120.27		—^d^	
**Engaged pages**									
	Full sample	0-202		6.92		80.33		4.60^e^	
	≥3 seconds	1-202		7.32		82.34		—	

^a^N=16,078.

^b^892/16,078.

^c^n=15,161.

^d^Not applicable.

^e^740/16,078.

The WLSMV estimator uses pairwise deletion for missing data; the first and second models used data from 99.5% (16,003/16,078) and 93.8% (15,086/16,078) of the full analytic sample, respectively. The analytic samples exceeded the recommended sample size of 300 for CFA [42]. While there are no absolute standards for an adequate sample size for SEM, the sample exceeded recommendations of a sample size of 10-20 per estimated parameter or a range from 200 to 1000 observations for WLSMV estimation depending on model complexity [43-47].

There is evidence that traditional cutoffs for model fit based on the maximum likelihood estimator (eg, standardized root mean squared residual [SRMR] <0.08, comparative fit index [CFI] ≥0.95, and root mean square error of approximation [RMSEA] <0.06) may not be appropriate for models with censored, ordinal, and dichotomous variables, and alternative indices of fit are being explored in the SEM literature [48-52]. However, to the best of our knowledge, there is no well-accepted method for interpreting model fit for the WLSMV estimator. For the purposes of this analysis, we reported traditional model fit indices and used traditional interpretations.

Theoretically consistent model modifications informed by Mplus MODINDICES outputs were conducted as exploratory analyses to determine whether model fit could be improved. Planning was removed from the full models such that intention predicted engagement. Next, intention and planning were removed such that the motivational HAPA constructs (ie, self-efficacy, perceived need, perceived risk, and outcome expectancies) predicted engagement. Descriptive data were analyzed in R (version 4.3.2; R Core Team, 2023) [53], and SEMs were conducted using Mplus (version 8.4; Muthén & Muthén) [54].

## Results

### Descriptive Statistics

The analytic samples included data from 16,078 sessions and a subsample of sessions in which participants spent at least 3 seconds on MHA outside of screening pages (n=15,161). See Table 2 for demographic characteristics. Correlations between the 22 Next Steps survey items, EM, and EP are shown in Multimedia Appendix 2.

**Table 2:** Demographic characteristics.

		Total sample (N=16,078)		<3 Seconds engagement (n=917)		≥3 Seconds engagement (n=15,161)	
**Age (years), n** **(%)**							
	11-17		2592 (16.1)		150 (16.4)		2442 (16.1)
	18-24		4323 (26.9)		129 (14.1)		4194 (27.7)
	25-34		2582 (16.1)		91 (9.9)		2491 (16.4)
	35-44		1099 (6.8)		41 (4.5)		1058 (7)
	45-54		515 (3.2)		21 (2.3)		494 (3.3)
	55-64		262 (1.6)		8 (0.9)		254 (1.7)
	≥65		90 (0.6)		6 (0.7)		84 (0.6)
	More than 1 answer given^a^		125 (0.8)		1 (0.1)		124 (0.8)
	Missing		4490 (27.9)		470 (51.3)		4020 (26.5)
**Race and ethnicity, n** **(%)**							
	American Indian or Alaska Native		133 (0.8)		7 (0.8)		126 (0.8)
	Asian		2088 (13)		85 (9.3)		2003 (13.2)
	Black or African American (non-Hispanic)		772 (4.8)		39 (4.3)		733 (4.8)
	Hispanic or Latino		962 (6)		28 (3.1)		934 (6.2)
	Middle Eastern or North African		378 (2.4)		20 (2.2)		358 (2.4)
	More than 1 of the above		427 (2.7)		11 (1.2)		416 (2.7)
	Native Hawaiian or other Pacific Islander		41 (0.3)		3 (0.3)		38 (0.3)
	Other		574 (3.6)		27 (2.9)		547 (3.6)
	White (non-Hispanic)		5631 (35)		208 (22.7)		5423 (35.8)
	More than 1 answer given		174 (1.1)		3 (0.3)		171 (1.1)
	Missing		4898 (30.5)		486 (53)		4412 (29.1)
**Gender identity^b^, n (%)**							
	Another gender identity		502 (3.1)		24 (2.6)		478 (3.2)
	Woman		8652 (53.8)		324 (35.3)		8328 (54.9)
	Man		2444 (15.2)		104 (11.3)		2340 (15.4)
	More than 1 answer given		76 (0.5)		2 (0.2)		74 (0.5)
	Nonbinary		2 (0.01)		0 (0)		2 (0.01)
	Missing		4402 (27.4)		463 (50.5)		3939 (26)
**Transgender, n** **(%)**							
	No		11102 (69.1)		430 (46.9)		10672 (70.4)
	Yes		176 (1.1)		11 (1.2)		165 (1.1)
	More than 1 answer given		461 (2.9)		13 (1.4)		448 (3)
	Missing		4339 (27)		463 (50.5)		3876 (25.6)
**Depression (PHQ-9)^c^, n (%)**							
	Minimal		139 (0.9)		1 (0.1)		138 (0.9)
	Mild		513 (3.2)		4 (0.4)		509 (3.4)
	Moderate		1041 (6.5)		15 (1.6)		1026 (6.8)
	Moderately severe		1666 (10.4)		11 (1.2)		1655 (10.9)
	Severe		2367 (14.7)		23 (2.5)		2344 (15.5)
	Missing		10,352 (64.4)		863 (94.1)		9489 (62.6)
**Anxiety (GAD-7)^d^, n (%)**							
	Minimal		157 (1)		1 (0.1)		156 (1)
	Mild		718 (4.5)		6 (0.7)		712 (4.7)
	Moderate		1298 (8.1)		10 (1.1)		1288 (8.5)
	Severe		2667 (16.6)		17 (1.9)		2650 (17.5)
	Missing		11,238 (69.9)		883 (96.3)		10,355 (68.3)

^a^“More than 1 answer given” includes participants who provided different responses in multiple sessions on Mental Health America.

^b^The original response options for gender identity included “female” and “male.” We report these as “woman” and “man”, respectively, to distinguish from sex assigned at birth.

^c^PHQ-9: Patient Health Questionnaire-9 [30].

^d^GAD-7: General Anxiety Disorder-7 [55].

### Measurement Models

Self-efficacy, outcome expectancies, intention, and planning originally had 5 categorical items corresponding to the 5 types of resources: LMH, CO, LTO, RMH, and ONL. The CO items had the lowest loadings on self-efficacy, outcome expectancies, and intention (β=.49-.55; *P*<.001). The CO item (“Do you have a specific plan to connect with others who have mental health conditions?”) was the third-lowest loading item for planning (β=.61; *P*<.001). To maintain consistency among the measurement models, all CO items were removed, and CFAs were conducted using the remaining 4 items (LMH, LTO, RMH, and ONL). Standardized coefficients and model fit indices for the final confirmatory 1-factor models are shown in Table 3. All standardized factor loadings of each latent construct were significant (*P*<.001).

**Table 3:** Standardized coefficients and model fit indices for measurement models.

Construct, items		Standardized λ^a^ (SE)	Chi-square (*df*)	*P* value	CFI^b^	RMSEA^c^ (90% CI)	SRMR^d^
**Self-efficacy**			613.4 (2)	<.001	0.99	0.14 (0.13-0.15)	0.02
	LMH^e^	0.78 (0.004)					
	LTO^f^	0.93 (0.003)					
	RMH^g^	0.74 (0.01)					
	ONL^h^	0.65 (0.01)					
**Outcome expectancies**			694.6 (2)	<.001	0.98	0.15 (0.14-0.16)	0.03
	LMH	0.69 (0.01)					
	LTO	0.92 (0.01)					
	RMH	0.77 (0.01)					
	ONL	0.55 (0.01)					
**Intention**			759.3 (2)	<.001	0.98	0.15 (0.14-0.16)	0.03
	LMH	0.68 (0.01)					
	LTO	0.93 (0.004)					
	RMH	0.76 (0.01)					
	ONL	0.53 (0.01)					
**Planning**			863.4 (2)	<.001	0.88	0.16 (0.16-0.17)	0.07
	LMH	0.53 (0.01)					
	LTO	0.995 (0.02)					
	RMH	0.47 (0.01)					
	ONL	0.45 (0.01)					

^a^λ: standardized regression coefficient; all coefficients were significant (*P*<.001).

^b^CFI: comparative fit index.

^c^RMSEA: root mean square error of approximation.

^d^SRMR: standardized root mean squared residual.

^e^LMH: learning more about mental health.

^f^LTO: learning about treatment options.

^g^RMH: receiving mental health treatment.

^h^ONL: using online self-help tools.

### Structural Models

#### Predicting Choice to Engage

**Figure 2:**
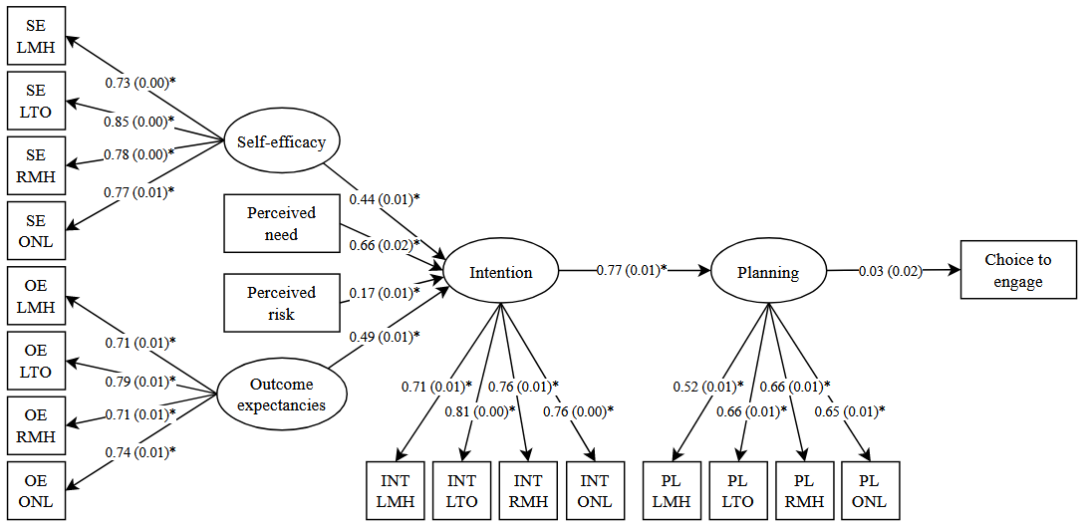
Structural equation model predicting choice to engage. Chi-square(147)=40,022.3, *P*<.001, comparative fit index=0.81, root mean square error of approximation=0.13, 90% CI 0.129-0.131, and standardized root mean square residual=0.14. *P<.001. INT: intention; LMH: learning more about mental health; LTO: learning about treatment options; OE: outcome expectancies; ONL: using online self-help tools; PL: planning; RMH: receiving mental health treatment; SE: self-efficacy.

The first model predicted whether participants chose to engage with the MHA website. The model comprised the HAPA constructs and a dichotomous outcome variable, choice to engage (Figure 2). Model fit was poor: *χ*^2^_147_=40,022.3, *P*<.001, CFI=0.81, RMSEA=0.13, 90% CI 0.129-0.131, and SRMR=0.14. Of note, the RMSEA is often inflated in models with small *df* and should be interpreted with caution [56]. The strongest predictor of intention was perceived need (β=.66, SE=0.02, *P*<.001), and intention significantly predicted planning (β=.77, SE=0.01, *P*<.001). However, choice to engage was not significantly predicted by planning (β=.03, SE=0.02, *P*=.18)*.* The model accounted for 83% of the variance in intention, 58.7% of the variance in planning, and 0.1% of the variance in choice to engage, although these estimates should be interpreted with caution due to poor model fit. See Table 4 for latent variable correlations.

**Table 4:** Latent variable correlations.

			Intention		Self-efficacy		Outcome expectancies		Planning
**Model 1: Predicting choice to engage, *r* (*P* value)**									
	Self-efficacy	0.77 (<.001)		—^a^		—		—	
	Outcome expectancies	0.79 (<.001)		0.68 (<.001)		—		—	
	Planning	0.77 (<.001)		0.59 (<.001)		0.61 (<.001)		—	
	Choice to engage	0.02 (.18)		0.02 (.18)		0.02 (.18)		0.03 (.18)	
**Model 2: Predicting level of engagement, *r* (*P* value)**									
	Self-efficacy	0.77 (<.001)		—		—		—	
	Outcome expectancies	0.79 (<.001)		0.67 (<.001)		—		—	
	Planning	0.77 (<.001)		0.59 (<.001)		0.60 (<.001)		—	
	Level of engagement	0.09 (<.001)		0.07 (<.001)		0.07 (<.001)		0.12 (<.001)	

^a^Correlations on the principal diagonal of the correlation matrix and redundant correlations are not shown.

#### Predicting the Level of Website Engagement

**Figure 3:**
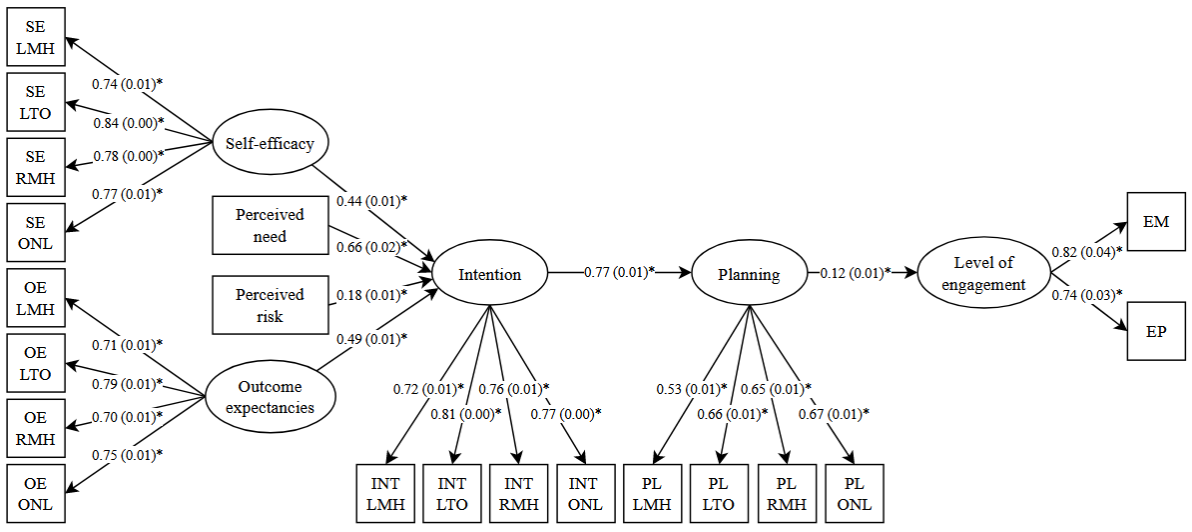
Structural equation model predicting level of engagement. Chi-square(164)=36,925.1, *P*<.001, comparative fit index=0.84, root mean square error of approximation=0.12, 90% CI 0.121-0.123, and standardized root mean square residual=0.14. *P<.001. EM: engaged minutes; EP: engaged pages; INT: intention; LMH: learning more about mental health; LTO: learning about treatment options; OE: outcome expectancies; ONL: using online self-help tools; PL: planning; RMH: receiving mental health treatment; SE: self-efficacy.

Using the same structural model, we predicted level of engagement within the subsample of participants who engaged with MHA for at least 3 seconds (Figure 3). Similar to the first model, model fit was poor: *χ*^2^_164_=36,925.1, *P*<.001, CFI=0.84, RMSEA=0.12, 90% CI 0.121-0.123, and SRMR=0.14. The second model accounted for 83% of the variance in intention and 58.6% of the variance in planning. While the association between planning and level of engagement was statistically significant (β=.12, SE=0.01, *P*<.001), the model was able to account for only 1.4% of the variance in level of engagement. See Table 4 for latent variable correlations.

### Sensitivity Analyses

The first sensitivity analysis was conducted by computing correlations between time spent on pages of different content types (navigation, condition, treatment, provider, connect, and DIY tool), the number of pages visited per content type, and the Next Step survey items. Time spent and number of pages visited by content type exhibited low correlations with the Next Steps survey items (*r*=–0.08 to 0.26). A second sensitivity analysis examined the second SEM predicting level of engagement with EM and EP winsorized at the 95th percentile. Model fit remained poor: *χ*^2^_154_=39,373.3, *P*<.001, CFI=0.82, RMSEA=0.12, 90% CI 0.121-0.123, and SRMR=0.14.

### Exploratory Analyses

Because planning had the worst model fit among the measurement models, it was removed from each structural model. The first exploratory analysis examined the model predicting choice to engage without planning (ie, intention predicted choice to engage). This model accounted for 88% of the variation in intention, but intention was not significantly associated with choice to engage (β=.01, SE=0.02, *P*=.41), *χ*^2^_86_=25,070.8, *P*<.001, CFI=0.86, RMSEA=0.14, 90% CI 0.133-0.136, and SRMR=0.13. In the second model, planning was removed such that intention predicted level of engagement. Intention significantly predicted level of engagement (β=.09, SE=0.01, *P*<.001), and the model accounted for 88% of the variance in intention and 0.8% of the variance in level of engagement, *χ*^2^_99_=23,252.1, *P*<.001, CFI=0.89, RMSEA=0.13, 90% CI 0.123-0.126, and SRMR=0.13.

Next, self-efficacy, perceived need, perceived risk, and outcome expectancies were modeled to directly predict the engagement outcomes. In the first model, perceived need (β=.11, SE=0.04, *P*=.01) and perceived risk (β=.10, SE=0.02, *P*<.001) were significant predictors of choice to engage, *χ*^2^_41_=8,015.8, *P*<.001, CFI=0.92, RMSEA=0.11, 90% CI 0.108-0.112, and SRMR=0.14. The path coefficients for self-efficacy (β=.04, SE=0.03, *P*=.20) and outcome expectancies (β=–.03, SE=0.03, *P*=.26) were not statistically significant. The model accounted for 0.9% of the variance in choice to engage. In the second model, level of engagement was significantly associated with perceived need (β=.18, SE=0.03, *P*<.001), perceived risk (β=.09, SE=0.01, *P*<.001), and self-efficacy (β=.08, SE=0.02, *P*<.001) but not outcome expectancies (β=–.01, SE=0.02, *P*=.62). Only 1.7% of the variance in level of engagement was accounted for by the model, *χ*^2^_50_=8,060.1, *P*<.001, CFI=0.94, RMSEA=0.10, 90% CI 0.101-0.105, and SRMR=0.13.

## Discussion

### Principal Findings

This study found limited support for the HAPA model in predicting engagement with web-based mental health resources, although the adapted HAPA model was useful for understanding and predicting self-reported motivations, intentions, and planning. We found support for the HAPA model in the motivational phase, with a large percentage of explained variance in health behavior intention (83%) and planning (59%). Self-efficacy, perceived need, perceived risk, and outcome expectancies significantly predicted intention to engage. This is consistent with a meta-analysis, which found strong support for the motivational phase of the HAPA model [13]. The results also highlight the importance of perceived need, the strongest predictor of intention, when investigating engagement with DMH. Although referenced in prior studies of the HAPA model, perceived need is not often included as a construct in HAPA studies, including the meta-analysis. Our results indicate that perceived need may be an important construct to regularly measure in future HAPA research.

Consistent with previous work [57-59], we found evidence of the intention-behavior gap in predicting DMH website engagement. Neither intention nor planning was a strong predictor of the engagement outcomes. Choice to engage was not associated with self-efficacy, outcome expectancies, intention, or planning. Latent variable correlations between level of engagement and self-efficacy (*r*=0.07), outcome expectancies (*r*=0.07), intention (*r*=0.09), and planning (*r*=0.12) were statistically significant, but effect sizes were trivial and should be viewed with caution because of the poor fit of the overall model and other limitations, such as the use of a self-report measure and measuring engagement using MHA’s available metadata (number of pages visited and time spent on MHA, excluding the final page viewed). However, these coefficients are aligned with meta-analytic results, which found coefficients less than 0.18 for all HAPA constructs in predicting behavioral outcomes [13]. A number of studies examining the HAPA framework have also found evidence supporting the preintention-intention-planning phases but limited or no support for the association between planning and behavior [60-62]. For example, previous work demonstrated that HAPA constructs predicted intention and planning but did not find evidence for planning predicting behavior (physical activity) across samples of middle-aged, physically inactive women, adults with obesity, and walking duration in older adults [60-62]. One study of a 2-week web-based intervention similarly found greater support for the motivational phase than the volitional phase, as well as stronger associations between self-reported motivational HAPA constructs and self-reported behavior but weaker associations with self-reported motivational constructs and objective measures of engagement [14].

Assuming that our estimates in predicting DMH engagement are correct, we suspect that 1 reason our study had weaker estimates than those found in the meta-analysis may have been our use of objective measures of behavior rather than self-report outcomes. The stronger associations at the motivational phase of the model in our study and in the meta-analysis may be inflated by method bias, as all measures were self-reported except for engagement, though the context of DMH may also have played a role. Only 3 studies in the meta-analysis included objective measures of behavior, none focused on DMH, and none focused on extremely short time lags for measuring behavior [13]. While our findings align with those in another study that used an objective measure of engagement [14], the relative lack of objective measures in existing HAPA studies limits our ability to contextualize our findings within the larger HAPA literature. Due to this, it is unknown whether the lack of association with engagement in our study as compared with other studies was the result of the specific context of a mental health resource website, the use of an objective measure of behavior, or the short time lag of measurement.

These findings have important implications for researching and enhancing user engagement with self-guided mental health websites. Studies on various DMH forms, such as web-based interventions or digitally delivered psychotherapy, consistently show that real-world engagement is poorer than in controlled lab experiments. Future studies could focus on understanding the factors influencing small-scale engagement actions, such as split-second decisions to click on website resources. These actions may collectively contribute to more substantial, long-term health behaviors. We suspect that website design, specifically strategies ensuring easy access and appeal of resources aligned with user needs, interests, and identity [63], may be a better predictor of health behavior in this context than the HAPA model. The sheer volume of materials on websites such as MHA can be overwhelming, making the quality, relevance, and visual appeal of resource links potentially more influential on DMH website engagement than constructs such as self-efficacy and outcome expectancies [64]. Tailoring links based on information from screening and demographic surveys is being explored in our ongoing research through a randomized trial of personalized website design.

### Strengths and Limitations

Strengths of the study include a large real-world sample of participants across the United States seeking information on a broad spectrum of mental health conditions. Another strength was the application of SEM, a robust statistical technique, to a real-life DMH setting with a well-established theoretical basis for the hypothesized model. There has been little research on the mechanisms driving engagement with DMH websites, and to our knowledge, this study is the first to fill this gap in the literature by examining the HAPA model in the context of DMH website engagement.

This study has several limitations. The Next Steps survey, used as a self-report measure, is a proprietary tool that lacks psychometric evaluation and assesses simplified HAPA model constructs. Other studies of the HAPA model have distinguished, for example, between task self-efficacy, coping self-efficacy, and recovery self-efficacy, and between action planning and coping planning [16]. Nevertheless, this approach enabled the assessment of HAPA constructs in relation to the specific tools provided by the MHA website and aligns with the methodology of many other published HAPA studies, which also use study-specific measures without psychometric evaluation. In addition, this study did not examine these constructs across multiple time periods. The stability of these constructs over time in this context remains unexplored, leaving open the possibility that factors such as self-efficacy may fluctuate. However, our study assessed these constructs as closely as possible to the engagement behavior. Our measurement of engagement was limited to MHA’s metadata, focusing on pages visited and time spent, excluding time spent on the final page viewed. While this approach may not capture the full spectrum of engagement behaviors, it was the only feasible approach for gathering a very large number of responses in a real-world setting. This study relied on a convenience sample, as participants self-selected into the survey. This could introduce bias, limiting the generalizability of our findings. Moreover, the exploratory models were iteratively honed and are likely overfit to the data. However, the modified models also resulted in poor model fit, suggesting that the adapted HAPA model did not fit the data.

### Future Directions

Future research could address these limitations by using psychometrically sound measures, exploring temporal dynamics of HAPA constructs, broadening engagement assessments, and using more diverse participant recruitment strategies. The study design could be modified such that the HAPA constructs are measured over multiple time points to examine change or consistency in appraisal-motivational factors over time. Researchers may also explore other frameworks that might be more appropriate for understanding and enhancing engagement with DMH (eg, Social Norms Theory [65], Health Belief Model [66], and I-Change Model [67]). We encourage future researchers to continue to fill these gaps, especially by including objective measurements of behavioral outcomes and by conducting additional research across the wide array of DMH approaches beyond website usage.

### Conclusions

As online mental health services become increasingly popular, there is a growing need for standardized measures of online engagement with all DMH, including mental health resource websites such as MHA. Despite the recent proliferation of DMH tools, they are underutilized by the communities they intend to serve, and few studies have examined factors impacting DMH engagement and how it can be optimized. This study aimed to identify whether and which HAPA constructs (self-efficacy, perceived need, perceived risk, outcome expectancies, intention, and planning) were significant predictors of DMH engagement. Despite some evidence supporting the motivational phase of the HAPA model in this context, the key finding was that there was insufficient support for the HAPA model in predicting engagement with DMH. The literature has yet to identify predictors of real-world DMH engagement, and doing so is an emerging and crucial research priority. It is possible that poor model fit was due in part to the study limitations. Researchers are encouraged to explore the HAPA model and other frameworks of health behaviors to identify predictors of DMH engagement. Understanding factors that optimize DMH engagement is urgently needed to inform evidence-based approaches toward tailoring DMH websites to better serve people seeking online mental health support.

## Appendix

Multimedia Appendix 1 Next Steps survey.

Multimedia Appendix 2 Item correlations.

## Abbreviations

CFA: confirmatory factor analysis
CFI: comparative fit index
CO: connecting with others
DMH: Digital Mental Health
EM: engagement minutes
EP: engaged pages
HAPA: Health Action Process Approach
LMH: learning more about mental health
LTO: learning about treatment options
MHA: Mental Health America
ONL: using online self-help tools
RMH: receiving mental health treatment
RMSEA: root mean square error of approximation
SEM: structural equation modeling
SRMR: standardized root mean squared residual
WLSMV: weighted least squares means and variance—adjusted
