# Supplementary material for: Application of an Adapted Health Action Process Approach Model to Predict Engagement With a Digital Mental Health Website: Cross-Sectional Study
Source: JMIR Hum Factors. 2024 Aug 7;11:e57082. doi: 10.2196/57082 (PMC11339574; doi:10.2196/57082)
Supplement: Multimedia Appendix 1 [file humanfactors_v11i1e57082_app1.doc]

| **Perceived Need** | 1. Do you believe you need to do something to improve your emotional or mental health? |
| --- | --- |
| **Perceived Risk** | 1. Do you think your emotional or mental health problems are at risk of getting worse if you aren't able to take action? |
| **Outcome Expectancies** |  |
| LMH^a^ | 1. How much do you think learning more about mental health would help improve your mental health? |
| CO^b^ | 1. How much do you think connecting with others who have mental health conditions would help improve your mental health? |
| LTO^c^ | 1. How much do you think learning about treatment options would help improve your mental health? |
| RMH^d^ | 1. How much do you think receiving mental health treatment would help improve your mental health? |
| ONL^e^ | 1. How much do you think using online self-help tools would help improve your mental health? |
| **Intention** |  |
| LMH | 1. Do you intend to learn more about mental health? |
| CO | 1. Do you intend to connect with others who have mental health conditions? |
| LTO | 1. Do you intend to learn about treatment options? |
| RMH | 1. Do you intend to receive treatment for your mental health? |
| ONL | 1. Do you intend to use online self-help tools? |
| **Self-efficacy** |  |
| LMH | 1. How confident are you in your ability to learn more about mental health? |
| CO | 1. How confident are you in your ability to connect with others who have mental health conditions? |
| LTO | 1. How confident are you in your ability to learn about treatment options? |
| RMH | 1. How confident are you in your ability to receive treatment for your mental health? |
| ONL | 1. How confident are you in your ability to use online self-help tools? |
| **Planning** |  |
| LMH | 1. Do you have a specific plan to learn more about mental health? |
| CO | 1. Do you have a specific plan to connect with others who have mental health conditions? |
| LTO | 1. Do you have a specific plan to learn more about treatment options? |
| RMH | 1. Do you have a specific plan to receive treatment for your mental health? |
| ONL | 1. Do you have a specific plan to use online self-help tools? |

^a^LMH: learning more about mental health.

^b^CO: connecting with others.

^c^LTO: learning about treatment options.

^d^RMH: receiving mental health treatment.

^e^ONL: using online self-help tools.
