# Supplementary material for: Application of an Adapted Health Action Process Approach Model to Predict Engagement With a Digital Mental Health Website: Cross-Sectional Study
Source: JMIR Hum Factors. 2024 Aug 7;11:e57082. doi: 10.2196/57082 (PMC11339574; doi:10.2196/57082)
Supplement: Multimedia Appendix 2 [file humanfactors_v11i1e57082_app2.doc]

|  | 1 | 2 | 3 | 4 | 5 | 6 | 7 | 8 | 9 | 10 | 11 | 12 | 13 | 14 | 15 | 16 | 17 | 18 | 19 | 20 | 21 | 22 | 23 |
| --- | --- | --- | --- | --- | --- | --- | --- | --- | --- | --- | --- | --- | --- | --- | --- | --- | --- | --- | --- | --- | --- | --- | --- |
|  |  |  |  |  |  |  |  |  |  |  |  |  |  |  |  |  |  |  |  |  |  |  |  |
| 1. PL^a^ LMH^b^ |  |  |  |  |  |  |  |  |  |  |  |  |  |  |  |  |  |  |  |  |  |  |  |
| 2. PL CO^c^ | .47 |  |  |  |  |  |  |  |  |  |  |  |  |  |  |  |  |  |  |  |  |  |  |
| 3. PL LTO^d^ | .50 | .40 |  |  |  |  |  |  |  |  |  |  |  |  |  |  |  |  |  |  |  |  |  |
| 4. PL RMH^e^ | .12 | .31 | .54 |  |  |  |  |  |  |  |  |  |  |  |  |  |  |  |  |  |  |  |  |
| 5. PL ONL^f^ | .42 | .38 | .39 | .10 |  |  |  |  |  |  |  |  |  |  |  |  |  |  |  |  |  |  |  |
| 6. PN^g^ | .02 | .04 | .26 | .36 | .08 |  |  |  |  |  |  |  |  |  |  |  |  |  |  |  |  |  |  |
| 7. PR^h^ | -.08 | .06 | .11 | .28 | -.09 | .38 |  |  |  |  |  |  |  |  |  |  |  |  |  |  |  |  |  |
| 8. OE^i^ LMH | .37 | .22 | .24 | .19 | .23 | .25 | .09 |  |  |  |  |  |  |  |  |  |  |  |  |  |  |  |  |
| 9. OE CO | .21 | .51 | .16 | .15 | .12 | .19 | .08 | .44 |  |  |  |  |  |  |  |  |  |  |  |  |  |  |  |
| 10. OE LTO | .20 | .21 | .37 | .35 | .13 | .33 | .19 | .62 | .45 |  |  |  |  |  |  |  |  |  |  |  |  |  |  |
| 11. OE RMH | .07 | .16 | .30 | .45 | .04 | .41 | .28 | .47 | .37 | .73 |  |  |  |  |  |  |  |  |  |  |  |  |  |
| 12. OE ONL | .24 | .21 | .20 | .08 | .53 | .17 | -.02 | .49 | .34 | .45 | .38 |  |  |  |  |  |  |  |  |  |  |  |  |
| 13. INT^j^ LMH | .46 | .30 | .34 | .27 | .27 | .27 | .15 | .60 | .36 | .49 | .40 | .42 |  |  |  |  |  |  |  |  |  |  |  |
| 14. INT CO | .24 | .72 | .18 | .22 | .13 | .11 | .09 | .29 | .64 | .31 | .26 | .26 | .43 |  |  |  |  |  |  |  |  |  |  |
| 15. INT LTO | .23 | .24 | .50 | .51 | .16 | .38 | .24 | .44 | .29 | .62 | .56 | .34 | .62 | .38 |  |  |  |  |  |  |  |  |  |
| 16. INT RMH | .07 | .19 | .39 | .69 | .03 | .42 | .32 | .37 | .26 | .51 | .58 | .24 | .47 | .34 | .74 |  |  |  |  |  |  |  |  |
| 17. INT ONL | .27 | .24 | .26 | .15 | .66 | .21 | .03 | .39 | .25 | .34 | .26 | .69 | .47 | .30 | .45 | .36 |  |  |  |  |  |  |  |
| 18. SEF^k^ LMH | .28 | .20 | .24 | .22 | .23 | .21 | -.01 | .42 | .24 | .37 | .32 | .34 | .51 | .22 | .41 | .32 | .34 |  |  |  |  |  |  |
| 19. SEF CO | .15 | .55 | .11 | .13 | .10 | .05 | -.04 | .30 | .46 | .28 | .21 | .25 | .31 | .61 | .27 | .23 | .23 | .48 |  |  |  |  |  |
| 20. SEF LTO | .20 | .17 | .39 | .36 | .19 | .25 | .05 | .41 | .23 | .47 | .43 | .34 | .47 | .22 | .59 | .49 | .36 | .72 | .46 |  |  |  |  |
| 21. SEF RMH | .12 | .17 | .31 | .55 | .11 | .28 | .10 | .40 | .23 | .45 | .47 | .29 | .40 | .24 | .55 | .67 | .30 | .52 | .41 | .71 |  |  |  |
| 22. SEF ONL | .23 | .17 | .19 | .08 | .60 | .15 | -.09 | .37 | .21 | .29 | .22 | .60 | .38 | .19 | .32 | .22 | .69 | .56 | .36 | .56 | .47 |  |  |
| 23. EM^l^ | .03 | .02 | .07 | .08 | .07 | .12 | .07 | .03 | .03 | .04 | .05 | .06 | .08 | .02 | .07 | .07 | .07 | .07 | -.02 | .06 | .04 | .05 |  |
| 24. EP^m^ | .03 | .02 | .08 | .09 | .06 | .13 | .10 | .02 | .03 | .04 | .06 | .05 | .07 | .01 | .08 | .08 | .06 | .05 | -.04 | .06 | .03 | .05 | .64 |

^a^PL: planning.

^b^LMH: learning more about mental health.

^c^CO: connecting with others.

^d^LTO: learning about treatment options.

^e^RMH: receiving mental health treatment.

^f^ONL: using online self-help tools.

^g^PN: perceived need.

^h^PR: perceived risk.

^i^OE: outcome expectancies.

^j^INT: intention.

^k^SEF: self-efficacy.

^l^EM: engaged minutes.

^m^EP: engaged pages.
